# Online Adaptive MR-Guided Ultrahypofractionated Radiotherapy of Prostate Cancer on a 1.5 T MR-Linac: Clinical Experience and Prospective Evaluation

**DOI:** 10.3390/curroncol31050203

**Published:** 2024-05-09

**Authors:** Vlatko Potkrajcic, Cihan Gani, Stefan Georg Fischer, Simon Boeke, Maximilian Niyazi, Daniela Thorwarth, Otilia Voigt, Moritz Schneider, David Mönnich, Sarah Kübler, Jessica Boldt, Elgin Hoffmann, Frank Paulsen, Arndt-Christian Mueller, Daniel Wegener

**Affiliations:** 1 Department of Radiation Oncology, University Hospital Tübingen, 72076 Tuebingen, Germany; 2 Department of Radiation Oncology, Klinikum Esslingen, 73730 Esslingen am Neckar, Germany; 3 Section for Biomedical Physics, Department of Radiation Oncology, University Hospital Tübingen, 72076 Tuebingen, Germany; 4 Department of Radiation Oncology and Radiotherapy, RKH-Kliniken Ludwigsburg, 71640 Ludwigsburg, Germany; 5 Department of Radiation Oncology, Alb-Fils Kliniken GmbH, 73035 Goeppingen, Germany

**Keywords:** prostate cancer, hypofractionated radiotherapy, MR-Linac

## Abstract

The use of hypofractionated radiotherapy in prostate cancer has been increasingly evaluated, whereas accumulated evidence demonstrates comparable oncologic outcomes and toxicity rates compared to normofractionated radiotherapy. In this prospective study, we evaluate all patients with intermediate-risk prostate cancer treated with ultrahypofractionated (UHF) MRI-guided radiotherapy on a 1.5 T MR-Linac within our department and report on workflow and feasibility, as well as physician-recorded and patient-reported longitudinal toxicity. A total of 23 patients with intermediate-risk prostate cancer treated on the 1.5 T MR-Linac with a dose of 42.7 Gy in seven fractions (seven MV step-and-shoot IMRT) were evaluated within the MRL-01 study (NCT04172753). The duration of each treatment step, choice of workflow (adapt to shape-ATS or adapt to position-ATP) and technical and/or patient-sided treatment failure were recorded for each fraction and patient. Acute and late toxicity were scored according to RTOG and CTC V4.0, as well as the use of patient-reported questionnaires. The median follow-up was 12.4 months. All patients completed the planned treatment. The mean duration of a treatment session was 38.2 min. In total, 165 radiotherapy fractions were delivered. ATS was performed in 150 fractions, 5 fractions were delivered using ATP, and 10 fractions were delivered using both ATS and ATP workflows. Severe acute bother (G3+) regarding IPS-score was reported in five patients (23%) at the end of radiotherapy. However, this tended to normalize and no G3+ IPS-score was observed later at any point during follow-up. Furthermore, no other severe genitourinary (GU) or gastrointestinal (GI) acute or late toxicity was observed. One-year biochemical-free recurrence survival was 100%. We report the excellent feasibility of UHF MR-guided radiotherapy for intermediate-risk prostate cancer patients and acceptable toxicity rates in our preliminary study. Randomized controlled studies with long-term follow-up are warranted to detect possible advantages over current state-of-the-art RT techniques.

## 1. Introduction

Prostate cancer is the most common cancer diagnosed in men [1] and the fifth leading cause of death worldwide [2]. Radiotherapy (RT) represents one of the standard treatment options for patients with localized disease [1]. Advances in technology and a better understanding of the biology of the disease have improved the treatment options [3] and allowed the implementation of hypofractionated and ultrahypofractionated (UHF) RT regimens [4,5]. The linear–quadratic model and biological features of prostate cancer, with an estimated α/β of about 1.5 Gy, suggest an improved therapeutic effect if larger doses per fraction are applied [4,6]. Thus, due to its high radiation-fraction sensitivity of prostate cancer, the use of hypofractionated RT has been increasingly encouraged [7]. Compared to normofractionated therapy over eight weeks, accumulating evidence demonstrates non-inferior results of (ultra)hypofractionated regimens regarding oncologic outcomes, without an increase in late toxicity rates [7,8,9,10]. Moderate hypofractionated RT is typically delivered in twenty fractions, whereas UHF RT usually consists of five to seven sessions (with >5 Gy per fraction) [7,8]. 

MRI-guided radiotherapy (MRgRT) using hybrids of a linear accelerator and a magnetic resonance scanner (MR-linear accelerator or MR-Linac) has improved the field of adaptive treatment and image-guided radiotherapy (IGRT) and therefore has been rapidly implemented into radiotherapeutical treatment options for various treatment indications [11,12,13]. The goal of this technology is to visualize all anatomical changes during the course of therapy, using MR imaging that enables superior visibility of soft tissue (including the prostate capsule), with an ability to adapt the treatment to given anatomical changes [12] using daily online MR imaging and plan adaptation, as well as online motion monitoring during radiotherapy [13,14]. Existing data on MR-guided UHF RT of prostate cancer report significantly lower acute toxicity of MR-guided treatment, compared to conventional UHF RT [15]. Furthermore, accumulating evidence suggests low clinician-scored late toxicity rates following UHF RT on MR-Linac [16,17,18,19].

The aim of our study was to report on all consecutive and prospectively enrolled patients treated at our institution using MR-guided online adaptive UHF RT for intermediate-risk prostate cancer. We assessed the feasibility concerning completion of treatment, treatment workflow, toxicity, and oncologic outcomes.

## 2. Materials and Methods

Patients with prostate cancer were enrolled in the prospective single-institution MRL-01 basket study (ClinicalTrials.gov Identifier: NCT04172753). The study protocol was approved by the local Ethics Committee (Nr. 659/2017BO1). Included were patients with histologically confirmed node-negative low- or intermediate-risk prostate cancer that were eligible for UHF treatment with RT on MR-Linac (prostate volume < 80 mL, IPS-score < 15). Patients with contraindications for MRI were excluded (metal implants, pacemakers, severe tinnitus, or claustrophobia). Risk stratification was performed according to current National Comprehensive Cancer Network (NCCN) guidelines for prostate cancer (version 4.2019) [20].

RT was delivered using 42.7 Gy (ICRU) in 7 fractions (6.1 Gy per fraction) 3 days per week, according to Widmark et al. 2019 [7]. RT planning was based on a planning CT (Big Bore RT, Philips, Amsterdam, The Netherlands; 3 mm slice thickness) and fused 2 min T2-weighted planning MRI sequence on the 1.5 T MRL (Unity, Elekta, Stockholm, Sweden; 2 mm slice thickness, isotropic). Target volume definition was performed using Monaco planning system v.5.51.11 (Elekta AB, Stockholm, Sweden). Clinical target volume (CTV) and organs at risk (OARs) were delineated following ESTRO ACROP guidelines [21]. Planning target volume (PTV) margins were generated using a 5 mm margin posteriorly and 6 mm in all other directions. Target volumes and OARs were initially contoured on the planning CT, aided by the information of the fused initial planning MRI T2-sequence. 

Treatments were delivered on a 7 MV MR-Linac using nine beam angles and step-and-shoot intensity-modulated RT (IMRT). For daily online adaptation, a 2-min T2-weighted MRI sequence was acquired. Two different types of plan adaptation are available at/for the MR-Linac Unity: The “adapt to shape” (ATS) adaptation is fully online-adaptive. Operators can modify all contours relevant for plan optimization based on the daily MR image, i.e., organs at risk and target volumes. This is followed by a full re-optimization of the treatment plan for the daily anatomy. The “adapt to position” (ATP) workflow is used to correct for daily translations of the patient with respect to the planned linac isocenter position, which would be corrected by a couch shift on conventional linacs. Couch shifts cannot be performed on the Unity MR-Linac. Therefore, a virtual couch shift is created by adapting the treatment plan MLC segments and weightings. ATP is non-adaptive, as it operates on the patient anatomy contoured in the pre-treatment reference image. In this study, the standard plan adaptation method was ATS. Near the end of the adaptation phase, a verification MR image was acquired. If patient motion without significant anatomical changes was observed, ATP was used to adjust the ATS plan. ATP alone was used only rarely, when patient anatomy was almost identical to the reference plan conditions.

Institutional constraints were used as follows: D0.05 cc ≤ 42.7 Gy was allowed for urethra and bulb of penis; V42.7 Gy ≤ 2 ccm for the bladder; and V30 Gy ≤ 1% for the femoral head. The anorectum was contoured including anal canal and rectum (until sigmoid). The following constraints were allowed for the anorectum: V42.7 Gy ≤ 0.01 ccm; V38.4 Gy ≤ 15%, V32 Gy ≤ 35%, V28 Gy ≤ 45%, and circumferential Dmax < 22 Gy.

To reduce the risk of urinary obstruction during the course of treatment, all patients prophylactically received alpha-blockers (tamsulosin 0.4 mg/day for 4 weeks, beginning with the first day of RT). The use of additional androgen deprivation therapy was considered individually in patients with unfavorable intermediate-risk prostate cancer.

Data were prospectively assessed and abstracted by chart review. The duration of each consecutive RT session was recorded (including each step of the daily adaptation process). Furthermore, treatment interruptions were evaluated, both technical and/or patient-sided. Acute and late toxicity were scored according to RTOG and CTC V4.0 using IPS-score, ICIQ score, and NCI-PRO-CTCAE (CTC for urinary frequency, GU urgency, GU obstruction, hematuria, diarrhea, rectal bleeding, proctitis, GI incontinence and ulcer, and RTOG for GU and GI toxicity). Grade 1 (G1) was defined as mild toxicity, grade 2 (G2) as moderate toxicity, and grade 3 or more (G3+) as severe toxicity. For IPS-score, 1–7 was defined as mild bother (G1), 8–19 as moderate bother (G2), and 20–35 as severe bother (G3). Scores were evaluated using physician- and patient-reported questionnaires before the beginning of radiotherapy, at the last RT session, and consecutively during follow-up (3, 6, and 12 months after the end of RT). 

Overall survival (OS) and biochemical recurrence-free survival (BCR-FS) were evaluated per patient based on the follow-up and prostate-specific antigen (PSA) measures. OS and BCR-FS were defined as the time from the date of the end of radiation therapy until death for OS and to the date of the last PSA control for BCR-FS. To define PSA progression, American Society for Radiation Oncology`s Phoenix definition was used (nadir plus 2.0 ng/mL) [22]. Statistical analysis was performed with IBM SPSS Version 26 and Microsoft Excel 2019.

## 3. Results

### 3.1. Patient Characteristics and Oncological Outcome

A total of 23 patients with prostate cancer treated with UHF RT between 2021 and 2022 were included in the analysis. The median follow-up was 12.4 months (range 2.9–19.6). One-year OS and BCR-FS were 100%. The patient, tumor, and treatment characteristics are presented in Table 1. All patients had intermediate-risk prostate cancer. Additional ADT was given in seven (30.4%) patients (six with unfavorable- and one with favorable intermediate-risk prostate cancer), with a median duration of 6 months (range 4–12 months).

### 3.2. Treatment Feasibility, Specifications, and Workflow

**Figure 1:**
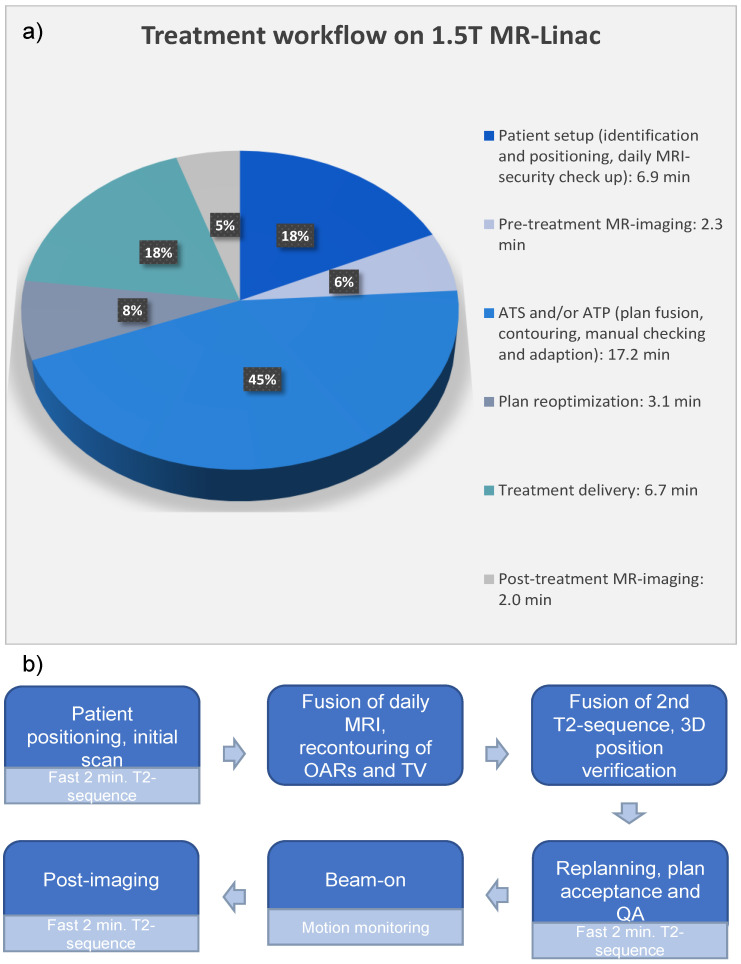
Panel (**a**). Pie chart demonstrating the mean time required for every step of the workflow for prostate cancer patients treated with online-adaptive UHF IGRT on the 1.5 T MR-Linac. The workflow starts with patient setup, followed by the steps listed in the clockwise rotation order. Mean total duration of each treatment session was 38.2 min. (MRI—magnetic resonance imaging, ATS—adapt to shape, ATP—adapt to position). Panel (**b**). Workflow graphic (OARs—organs at risk, TV—target volume, QA—quality assessment).

All patients (n = 23) completed the planned treatment. A total of 165 RT fractions (including completion plans) were applied on the 1.5 T MR Linac. The mean duration of a single treatment session (on-table time) for all patients was 38.2 min per fraction (range 33.3–43.4 min). Out of this time, the mean time of treatment delivery was 6.7 min, whereas a mean of 17.2 min was spent on contouring and plan adaptation. In 16 patients, treatment was delivered using the ATS workflow exclusively (total 112 fractions). In seven patients, the treatment was delivered using ATS and/or ATP (total 53 fractions). Out of these, most fractions were delivered using ATS (n = 38); in ten fractions, additional ATP was performed on top of the ATS workflow to account for motion observed in a verification image after plan adaption/before the start of treatment. Five fractions were delivered using only ATP. Due to technical reasons, five treatment interruptions were documented. For these fractions, completion plans were delivered as an additional eight or ninth radiotherapy session. No patient-related treatment failures were documented. The workflow of treatment delivery on MR-Linac is demonstrated in Figure 1.

### 3.3. Toxicity

An increase in IPS-score was reported at the end of RT (45%, 32%, and 23% for mild, moderate, and severe symptoms), compared to baseline score (70%, 26%, and 0%), mean IPS-score 10.4 vs. 5.7, *p* = 0.006. Subsequently, a significant decline was observed during follow-up (62%, 31%, and 0% at 3 months, 73%, 27%, and 0% at 12 months), mean 7.4 and 6.9, *p* = 0.015 (compared to end of radiotherapy). IPSS-related severe bother was reported in five patients (23%) at the end of RT; however, during follow-up (at 3, 6, and 12 months), no severe toxicity was reported. The ICIQ-score at baseline was 9%, 9%, and 0% for mild, moderate, and severe symptoms. Slightly increased moderate toxicity was observed at the end of RT (0%, 23%, and 0%). During follow-up, an ICIQ-score of 23%, 5%, and 0% at 3 months and 27%, 18%, and 0% at 12 months was reported.

**Figure 2:**
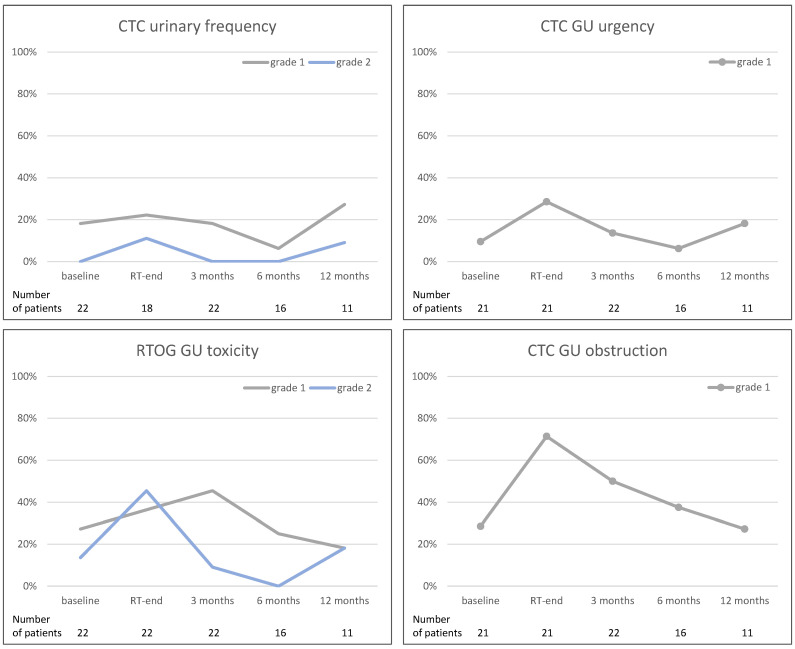
Acute genitourinary toxicity scored as CTC urinary frequency, urgency, and obstruction, as well as RTOG GU toxicity. Line graphs demonstrate number of patients in percent (y-axis) reporting the toxicity scores at the given point of time (x-axis). (GU—genitourinary, CTC—common toxicity criteria, RTOG—Radiation Therapy Oncology Group, RT—Radiotherapy).

Genitourinary toxicity, reported as CTC urinary frequency, urgency, and obstruction, as well as RTOG GU toxicity, are demonstrated in Figure 2. No G3+ toxicity was observed. For GU urgency and obstruction, a maximum of G1 toxicity was scored. For CTC urinary frequency and RTOG GU toxicity, a maximum of G2 toxicity was observed. Regarding CTC hematuria toxicity, G1 toxicity was reported at the end of RT in one patient; otherwise, no toxicity was reported at baseline and during the follow-up. Furthermore, a slight increase in G1 and/or G2 toxicity rates was scored 12 months after the end of RT (for CTC urinary frequency, CTC GU urgency, and RTOG GU toxicity).

**Figure 3:**
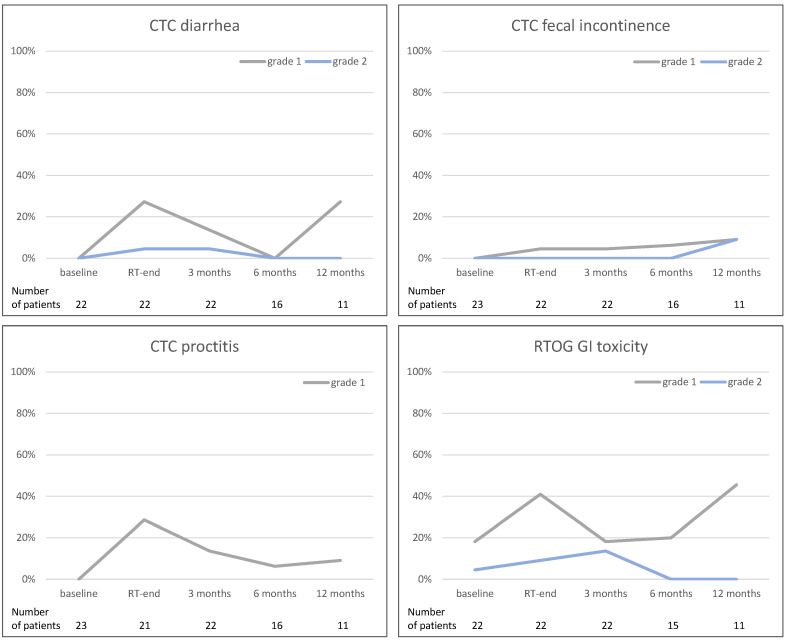
Acute gastrointestinal (GI) toxicity scored as CTC diarrhea, fecal incontinence, and proctitis, as well as RTOG GI toxicity. Line graphs demonstrate number of patients in percent (y-axis) reporting the mean toxicity scores at the given point of time (x-axis). (GI—gastrointestinal, CTC—common toxicity criteria, RTOG—Radiation Therapy Oncology Group, RT—Radiotherapy).

Gastrointestinal toxicity reported as CTC diarrhea, fecal incontinence and proctitis, as well as RTOG GI toxicity are demonstrated in Figure 3. No G3+ toxicity was observed. For CTC diarrhea and RTOG GI toxicity, from 6 months after the end of RT onwards, no patients reported on any G1+ toxicity. One patient reported grade 1 rectal bleeding at the end of RT and at 3 months post-RT, whereas no rectal bleeding was scored as late toxicity at further follow-up. Regarding rectal ulcers, two patients reported on toxicity grade 1 at 12 months. Furthermore, a slight increase in G1 and/or G2 toxicity rates was demonstrated 12 months after the end of RT (CTC diarrhea, CTC proctitis, CTC fecal incontinence, RTOG GI toxicity).

## 4. Discussion

This prospective study reports on the first results of all consecutive patients with intermediate-risk prostate cancer treated with MR-guided online adaptive UHF RT (42.7 Gy in 7 fractions) on a 1.5 T MR-Linac in our institution.

Widmark et al. reported on the 5-year results of a prospective phase III trial using UHF CT-guided radiotherapy with 42.7 Gy in seven fractions, establishing this fractionation regime. Equivalent failure-free survival rates of 84% at 5 years for both UHF and normofractionated regimen were demonstrated. However, the study presented a tendency for increased clinician-scored toxicity in the UHF group (increased acute urinary frequency of RTOG grade 2+ of 28% vs. 23%, *p* = 0.057, as well as one-year urinary toxicity of 6% vs. 2%, *p* = 0.003). Increased patient-reported GU and GI toxicity were demonstrated. However, no significant difference was reported at 3 months after the treatment [7]. 

Alongi et al. presented data on 100 patients treated in Brescia with UHF RT on a 1.5 T MR-Linac, with a similar follow-up of 12 months. The group reports a clinician-scored toxicity of three G2 and one G3 GU late event and three G2+ GI events, as well as excellent BFS rates [16]. Recently, Pridan et al. added data on 200 patients treated in Israel on a 0.35 T MR-Linac in five fractions, similarly reporting low clinician-scored toxicity rates (maximal CTC late GI and GU G2 toxicity in ca. 5% and 1.5% patients, respectively) and excellent outcomes after a mean follow-up of 16 months [18]. Tetar et al. and Bruynzeel et al. reported on 101 patients treated with UHF RT on MR-Linac using 36.25 Gy delivered in five fractions and also reported clinician-scored and patient-reported outcomes. The maximum cumulative early CTC G2+ GU and GI toxicity was 23.8% and 5.0%, respectively. With longer follow-up, clinician-reported G2+ GU toxicity remained 3.1%-5.1%, while G2+ GI toxicity was not observed. No G3+ GU or GI toxicity according to the CTCAE criteria was observed at any time point [17,19]. Furthermore, the PACE-B trial by Tree et al. reported on similar 2-year RTOG G2 toxicity rates in patients with low- or intermediate-risk PC treated with 36.25 Gy in 5 fractions, compared to patients treated with 78 Gy in 39 fractions or 62 Gy in 20 fractions [23].

The MIRAGE Trial by Kishan et al. is the only randomized clinical trial comparing MR-guided to conventional CT-guided UHF RT of prostate cancer, resulting in significantly lower acute toxicity of MR-guided treatment (GU CTC grade 2+ 24.4% vs. 43.3%, *p* = 0.01; GI CTC grade 2+ 0% vs. 10.5%, *p* = 0.003). However, late toxicity results have not been presented. These results might be explained with the differences in PTV-margins being used, where 4 mm isotropical CTV-expansion was used for CT-guided RT, whereas 2 mm margins were used for MR-guided RT [15]. The PTV-margins used in this study differ from those used in our study. Having been one of the first centers worldwide to implement an MR-Linac and due to lacking data on optimal margins for this technique, we used the same PTV-margins as for radiotherapy on conventional Linacs. However, in line with the results of the MIRAGE Trial, reduced PTV-margins shall be considered for patients treated with MR-guided radiotherapy. However, long-term follow-up data are needed to confirm that smaller PTV-margins do not lead to higher biochemical recurrence rates. 

Our data add well to the above-mentioned literature: We report no G3+ toxicity, neither clinician-scored nor patient-reported (ICIQ, RTOG and CTC and Pro-CTCae), except for a G3 IPS-score at the end of RT (23% of the patients). However, a significant decrease in IPS-score was observed during the follow-up, and no further G2+ late toxicity was reported. Patient selection based on prostate volume and baseline urinary symptoms, as well as the prophylactic application of alpha-blockers, seem to be justified by these results.

The treatment feasibility in our study was excellent, with all 23 patients successfully completing all planned RT sessions. Of note, to the best of our knowledge, our cohort is the only one in the literature reporting on a seven-fraction MR-guided UHF RT, presenting similarly low rates of toxicity as the five-fraction regimes, regardless of the variety in applied margins in the published studies. In summary, MR-guided online adaptive UHF RT seems very promising regarding a reduction in acute and long-term toxicity for prostate cancer treatment, but further follow-up of the existing studies and prospective randomized controlled studies are needed to determine possible long-term toxicity after UHF regimens.

Furthermore, no biochemical recurrences or deaths were noted during the follow-up, resulting in 100% one-year OS and 100% BCR-FS. However, due to the limited number of patients and follow-up time (median follow-up of 12.4 months), these data must be regarded as preliminary.

The mean treatment time (37.9 min per fraction) seems slightly longer compared to the results published by Keizer et al., also for the 1.5 T MR-Linac (33 min) [24]. However, after excluding the patient setup time, the results seem comparable (34.4 min). Daily online-adaptive RT results in a significantly better target coverage compared to non-adapted plans [25]. Thus, most RT fractions in our study were delivered using ATS (150/165 fractions), confirming the need for daily online adaptation in clinical routine.

## 5. Conclusions

Excellent feasibility and no late G3+ toxicity were demonstrated in patients with localized prostate cancer treated in our institution with MR-guided online adaptive UHF radiotherapy (42.7 Gy in 7 fractions) on a 1.5 T MR-Linac. Larger prospective studies and longer follow-up are needed to further evaluate the benefit of MR-guided UHF therapy.

## Figures and Tables

**Table 1:** Patient, tumor, and treatment characteristics (n = 23).

Age (years), Median (range)	76 (57–84 years)
**Gleason-score**	
6	1 (4.4%)
7a	11 (47.8%)
7b	11 (47.8%)
**Tumor stage**	
cT1c	2 (8.7%)
cT2a	12 (52.2%)
cT2b	6 (26.1%)
cT2c	3 (13.0%)
**Risk stratification according to NCCN**	
Favorable intermediate risk	7 (30.4%)
Unfavorable intermediate risk	16 (69.6%)
**Staging imaging modalities**	
MRI	23 (100.0%)
WBBS	8 (34.8%)
CT	4 (17.4%)
PSMA PET-CT	1 (4.4%)
**PSA value prior to RT (ng/mL)**	
<10 ng/mL	19 (82.6%)
≥10 ng/mL	4 (17.4%)
**Additional ADT**	7 (30.4%)

n—number of patients, NCCN—National Comprehensive Cancer Network, MRI—magnetic resonance imaging, WBBS—whole body bone scintigraphy, CT—computed tomography, PSMA PET-CT—prostate-specific membrane antigen–positron emission tomography–CT, PSA—prostate-specific antigen, RT—radiotherapy, ADT—androgen deprivation therapy.

## Data Availability

The datasets used and analyzed during the current study are available from the corresponding author on reasonable request.
